# Natural Polymeric Hydrogels Encapsulating Small Molecules for Diabetic Wound Healing

**DOI:** 10.3390/gels9110867

**Published:** 2023-10-30

**Authors:** Elena Iulia Oprita, Andreea Iosageanu, Oana Craciunescu

**Affiliations:** National Institute of R&D for Biological Sciences, 296, Splaiul Independentei, 060031 Bucharest, Romania; andreea.iosageanu@incdsb.ro (A.I.); oana.craciunescu@incdsb.ro (O.C.)

**Keywords:** hydrogel, flavonoids, collagen, alginate, cellulose, wound healing, diabetes

## Abstract

Diabetes is a condition correlated with a high number of diagnosed chronic wounds as a result of a complex pathophysiological mechanism. Diabetic chronic wounds are characterized by disorganized and longer stages, compared to normal wound healing. Natural polymer hydrogels can act as good wound dressings due to their versatile physicochemical properties, represented mainly by high water content and good biocompatibility. Natural bioactive hydrogels are polymers loaded with bioactive compounds providing antibacterial and antioxidant properties, modulation of inflammation and adherence to wounded tissue, compared to traditional dressings, which enables promising future applications for diabetic wound healing. Natural bioactive compounds, such as polyphenols, polysaccharides and proteins have great advantages in promoting chronic wound healing in diabetes due to their antioxidant, anti-inflammatory, antimicrobial, anti-allergic and wound healing properties. The present paper aims to review the wound healing mechanisms underlining the main issues of chronic wounds and those specifically occurring in diabetes. Also, the review highlights the recent state of the art related to the effect of hydrogels enriched with natural bioactive compounds developed as biocompatible functional materials for improving diabetic-related chronic wound healing and providing novel therapeutic strategies that could prevent limb amputation and increase the quality of life in diabetic patients.

## 1. Introduction

Diabetes mellitus (DM) represents a group of chronic metabolic diseases characterized by defective insulin secretion and insulin resistance, which determines increased blood glucose, a condition called hyperglycemia. In addition, hyperglycemia is frequently found associated with various complications, such as diabetic neuropathy, retinopathy, cardiomyopathy and nephropathy, leading to loss of sensation in the lower extremities, blindness, renal failure, chronic wounds and lower extremity amputations [1].

One in ten adults is diabetic and just above half a billion people are living with this condition worldwide, according to the 10th edition IDF Diabetes Atlas [2]. It is estimated that by 2045 the number of individuals suffering from diabetes will increase to 783.2 million people [3]. With this increasing trend, the global costs of diabetes are also expected to rise. Research shows that the total economic burden of diabetes reached about $966 billion in 2021 and is expected to reach $1054 billion by 2045 [4]. Regarding regional distribution, the countries with the highest prevalence of diabetes are China, India, the United States, Indonesia and Mexico, of which India is the country with the highest mortality rate from this condition [5].

Chronic wounds associated with diabetes can be classified as foot, venous and pressure ulcers, with diabetic foot ulcer (DFU) being among the most common complications of patients with diabetes [6]. Due to loss of sensation in the lower extremities caused by diabetic neuropathy, patients may not feel heat or abrasions and, thus, they can get hurt. The wound becomes infected, which, in conjunction with the hyperglycemic microenvironment, disrupts the healing process [7]. DFUs affect 19–34% of patients with diabetes and up to 25% of cases require amputation [8]. After amputation, the wound becomes larger and more difficult to heal, which is the reason for diabetic foot ulcer having just about 50% mortality within 5 years of amputation [9].

Conventional treatment uses the “TIME” principle to treat chronic wounds. It includes tissue debridement, infection control, moisture balance and edges of the wound [3]. First of all, wound bed preparation is very important to facilitate chronic wound healing. Tissue debridement involves the removal of non-viable tissue from the wound bed, in order to allow the tissue to heal. If there are any signs of infection, they must be treated promptly, as pathogens cause tissue damage and increase inflammatory response. Moisture balance refers to ensuring a warm and moist environment that is required for healing, while wound edges assessment can indicate the progress of healing and confirms if the treatment is effective [10].

However, the current strategy in the management of diabetic wounds is not entirely effective, with almost a quarter of patients undergoing low limb amputations [8]. Therefore, tissue engineering strategies are urgently needed, including the development of hydrogels and other bioactive platforms for the restoration of skin integrity and the improvement of patients’ quality of life.

In contrast to sponges and conventional bandages that only cover the wound and absorb its exudate, natural polymeric hydrogels are considered highly suitable candidates for diabetic wound dressings due to their good biocompatibility and antibacterial properties [1]. They can provide a moist environment, a microporous structure ensuring a good milieu for gas exchange and nutrient transport and a suitable environment mimicking the composition and/or structure of the extracellular matrix (ECM) for cell proliferation and migration [11]. Moreover, hydrogels, encapsulating small molecules or drugs, such as polyphenols, growth factors, or antibiotics, can further augment the stimulation of wound healing [12]. Accordingly, they can facilitate the healing process of chronic wounds and reduce the likelihood of amputations.

This paper aims to review the potential of novel natural polymeric hydrogels encapsulating small molecules and the involved mechanisms to effectively manage the healing of diabetic wounds. First, the differences between the biological and biochemical processes in normal vs. diabetic wound healing were summarized. Recent studies on in vitro and in vivo effects of small molecules and natural gelling polymers were reviewed. The primary focus of this review lay in exploring the natural polymeric hydrogels transformed into delivery platforms for the administration of incorporated beneficial small molecules that can facilitate the complex process of diabetic wound healing. The ultimate objective of this comprehensive review was to offer scientific insights and guidance for further management of diabetic wounds with a special emphasis on advanced natural hydrogels for reducing the incidence of amputations in individuals suffering from diabetes.

## 2. Biological and Biochemical Processes during Normal vs. Diabetic Wound Healing

Skin lesions are classified as acute wounds occurring after injury or trauma and going through normal wound healing processes, and complex chronic wounds constantly affected by abnormal factors that prevent the unfolding of normal stages of the wound healing process. Thus, chronic wounds may stall in one particular stage of the healing process (e.g., inflammatory stage) for a longer time, which desynchronizes the cascade events, causing prolongation of the healing process, in particular in diabetic wounds [12].

### 2.1. Stages of Normal Wound Healing

**Figure 1:**
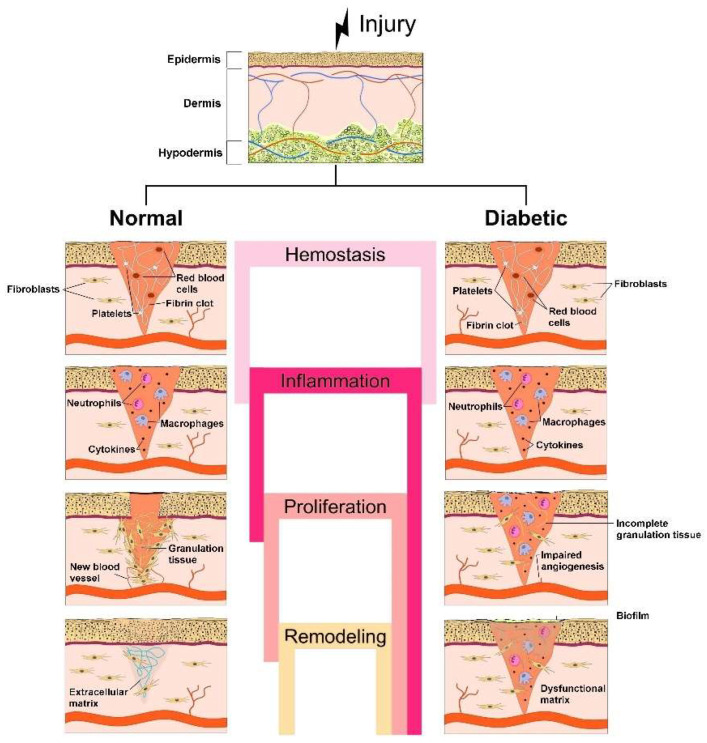
Schematic representation of the stages of normal wound healing, i.e., hemostasis, inflammation, proliferation and remodeling, vs. the pathogenesis of diabetic wounds, considering the chronic inflammatory phase that leads to incomplete granulation of tissue, impaired angiogenesis, formation of dysfunctional matrix and, finally, delaying the healing process.

Normal wound healing is a continuous and dynamic process that involves a multitude of biochemical interactions at the cellular and molecular levels. According to Singer and Clark [13], the process can be divided into four overlapping stages: hemostasis, inflammation, proliferation and remodeling (maturation) [14] (Figure 1). The involvement of particular cell types is required and varies during different stages of repair. There is also the need for growth factors, cytokines, regulatory molecules, enzymes and components of the ECM for repairing and restoring the damaged tissue, according to the injury degree [15].

The hemostasis stage (few hours) begins immediately after an injury to stop bleeding and is characterized by:

- Vascular constriction that decreases blood circulation at the wound site;
- Platelet aggregation at the wound site owing to the interaction with proteins (collagen (COL) and fibronectin);
- Degranulation;
- Conversion of soluble fibrinogen into insoluble fibrin to arrest bleeding;
- Mediation of hemostasis through key agents, such as fibrin, fibronectin and vitronectin;
- Production of growth factors, such as transforming growth factor β (TGF-β), platelet-derived growth factor (PDGF), fibroblast growth factor (FGF), epidermal growth factor (EGF) and chemokines, by the clot’s surrounding area to efficiently aid the wound healing [15,16]. PDGF acts also in vascularization by attracting fibroblasts, which stimulate tissue repair through COL deposition [17]. In the first phase of the homeostasis stage, prostaglandin H2 is converted into thromboxane A2 (TXA2) by the action of thromboxane synthase. Then, TXA2 acts as a powerful platelet activator and vasoconstrictor, in addition to participating in the release of macrophages, neutrophils and endothelial cells, playing an important role in the following stages of the wound healing process [18,19].

The inflammation stage (1–6 days), initiated when the bleeding has stopped, has the goal of avoiding the entrance of pathogens, preventing infections and more severe complications. This stage is characterized by:

- Leucocytes’ (especially neutrophils) migration to the injured site to eliminate debris and bacteria;
- Proinflammatory cytokines secretion by neutrophils to promote the expression of adhesion molecules;
- Monocytes’ migration into the wound site and differentiation into macrophages.

Neutrophils, monocytes and macrophages play an important role due to the release of proinflammatory cytokines, such as interleukin-1 (IL-1), IL-6, IL-8, tumor necrosis factor α (TNF-α) and growth factors (PDGF, TGF-α, TGF-β, FGF and insulin-like growth factor-1 (IGF-1)) involved in the activation of fibroblasts and epithelial cells needed in the proliferation stage [19,20].

The proliferation stage (epidermal regeneration) (4–21 days) diminishes the wound area by epidermal regeneration, granulation tissue formation, COL synthesis to provide tissue strength and angiogenesis [21]. The activated fibroblasts migrate to the wound bed and synthesize ECM constituents (hyaluronan, fibronectin and proteoglycans), leading to the replacement of the fibrin clot. The granulation tissue is a connective tissue in which the predominant protein is COL type III, which provides a scaffold for novel ECM and blood vessel synthesis. The process of re-epithelialization taking place in this stage creates a new epidermal barrier after keratinocytes’ proliferation and migration to the wound tissue mediated by cytokines secreted by innate immune cells [19]. Endothelial cell growth and chemotaxis are regulated by low oxygen tension and the production of lactic acid and biogenic amines. Furthermore, fibroblasts are differentiated into myofibroblasts, a process that results in lower cell proliferation together with an increase in COL synthesis [12]. In consequence, in this stage, wound contraction takes place to reduce it to a smaller size [19]. 

The remodeling (scar maturation) stage (3 weeks–2 years) is characterized by:

- Slow transformation of ECM into a mature scar;
- COL production reorganization in the ECM by replacing COL type III with COL type I and closure of the wound;
- Decrease of blood supply and formation of new blood vessels;
- Formation of a cellular environment and mature avascular tissue [19].

After all four stages of normal wound healing, it was reported that skin could only recover a maximum tensile strength of 80% [16].

### 2.2. Pathology of Diabetic Wound Healing

The primary factors hindering wound healing in diabetic patients do not deviate from those involved in the development of other chronic wounds. However, the underlying causes for the emergence of these factors differ. Diabetic wounds can evolve into chronic wounds due to a hyperglycemic environment that is ideal for bacterial growth and increases the risk of microbial infection. Untreated infections promote inflammation at the wound bed, as well as oxidative stress, both altering the wound healing process [9]. In addition, diabetic wounds become complicated to heal due to the following disturbing conditions: hypoxia, chronic inflammation leading to excessive expression of matrix metalloproteinases (MMPs), and impaired angiogenesis [22]. 

The main factor of diabetic wound damage is severe hypoxia occurring after tissue injury. Oxygen is very important in wound healing to promote fibroblast proliferation, enhance immune function and stimulate angiogenesis. Diabetic patients have an inadequate oxygen supply due to vascular dysfunction and neuropathy, and a high oxygen consumption rate by recruitment of inflammatory cells in the wounded area [23,24]. Thus, long-term oxygen deprivation impairs the wound healing process [24,25]. In a milieu with low blood oxygen levels, the expression of the hypoxia-inducible factor 1α (HIF-1α) gene and the synthesis of HIF-1α are lowered, the cell response is hindered, and the angiogenesis is affected, thus causing the wound healing process to be slowed down [16]. Also, hypoxia stimulates the inflammatory response and increases the level of free oxygen radicals, leading to an extension of the duration of effective wound healing [23,24]. 

Compared with normal wound healing, diabetic wound healing is characterized by a chronic inflammatory phase due to uncontrolled inflammation and the persistence of inflammatory cells at the wounded area (Figure 1). In chronic inflammation, a significant decrease of fibroblast proliferation, function and differentiation into myofibroblasts takes place, together with lowering of TGF-β type II receptor expression and COL synthesis, thus also hindering the final phase of skin remodeling [24,26]. In addition, macrophages and their phenotype play an important role in the transition between wound healing stages, in particular in the inflammatory stage. M1 macrophages have been defined as having an active role in the secretion of pro-inflammatory cytokines, such as TNF-α, IL-1, IL-6 and IL-8 during inflammation, while M2 macrophages were involved in anti-inflammatory activities during remodeling [18]. Macrophages’ transition from the M1 to M2 phenotype is modulated by activation of p38/MKP-1, microRNA-21 and protein kinase B (AKT) signaling pathways. In cases of this transition being blocked and the failure of M2 macrophages to be made available, the healing process is prevented, the remodeling stage is not reached and the entire wound healing process is endangered [18,20]. Besides, the inflammatory phase in diabetic wounds with high glucose levels results in increased expression of MMPs, liable for excessive ECM degradation and regulation of keratinocyte migration. The increase of GFs and GF receptors’ levels along with that of integrins and integrin receptors results in the slowing down of diabetic wound healing [24].

Diabetic wounds are, also, defined by a slow formation of new blood vessels at the wound situs that disturbs the blood supply. The mechanisms of this pathological process are correlated to bacterial contamination, imbalanced angiogenic factors, e.g., TGF-α, TGF-β, FGF-2, vascular endothelial growth factor (VEGF), EGF, HIF-1α, and vascular inhibitory factors, such as platelet-activating protein and vasopressors. Also, diabetics develop macroangiopathy, which leads to reduced blood flow. Thus, the production of NO decreases because of reduced levels of nitric oxide synthase (NOS), abnormal capillary regulation and impaired diastolic function of the vasculature, leading to microvascular malfunction [24]. Diabetic patients with sustained hyperglycemia states suffer alterations in the endothelial cells’ metabolism and function, which results in various defective micro- and macro-circulatory issues triggering a hindered angiogenesis process. A damaged vasculature obstructs oxygen delivery to the tissue, creating a hypoxic microenvironment and causing persistent inflammation at the wound situs [27]. Moreover, this microenvironment is able to impair endothelial cell function, thereby disrupting the balance between pro-angiogenic and anti-angiogenic factors, leading to reduced angiogenesis and creating a loop [11]. 

The healing time of diabetic wounds is longer due to an excessive and abnormal accumulation of several compounds like advanced glycation end products (AGEs) and reactive oxygen species (ROS), pro-/anti-inflammatory cytokines balance, interleukins, leukotrienes and complement factors at the wound site, but also to lack of ECM proteins [28]. The accumulation of AGEs can alter keratinocyte and fibroblast function. The formation of free oxygen radicals can produce harmful effects on blood supply and the structure and metabolism of peripheral nerves. Thus, in diabetic patients, hand–foot neuropathy is the most common form and one of the main reasons for impaired healing of foot wounds [16]. In diabetic foot ulcers and diabetic nephropathy, activators of nuclear factor erythroid 2-related factor 2 (Nrf2) reduce oxidative stress and improve the process of wound healing. The transcription factors Nrf2 and nuclear factor kappa B (NF-kB) promote wound healing through anti-inflammatory and antioxidant effects or via the immune system. They have a key and reciprocal role [29]. Nrf2, the primary regulator of intracellular redox homeostasis, is a redox-sensitive transcription factor, which regulates the expression of cytoprotective genes, leading to keratinocyte apoptosis, repair-related inflammation and protection against excessive accumulation of ROS. In turn, NF-kB activates the immune response, cell proliferation and migration, and regulates the expression of MMPs, secretion and the stability of cytokines and growth factors for wound healing [23,26].

## 3. Effect of Small Molecules and Natural Polymers in Diabetic Wound Healing

Plant-derived bioactive compounds or small molecules are mainly classified as polyphenols, triterpenes, carotenoids and tocopherols, alkaloids, glucosinolates and capsaicinoids. They manifest antioxidant, anti-inflammatory, antimicrobial, anti-allergic, antiproliferative and antimutagenic properties of interest for the human health. The structure–activity correlation allows their various applications as main ingredients of products for the food and pharmaceutical industry [30,31].

### 3.1. Small Molecules

Polyphenols, a large family of plant secondary metabolites, are characterized by the presence of a huge number of hydroxyl groups attached to aromatic rings and are mainly divided into flavonoid and non-flavonoid compounds [32]. It was demonstrated that phenolic compounds could provide useful properties in the wound healing process. Polyphenols modulate the inflammatory agents, such as cytokines, increase angiogenesis and vascular genesis, can promote epithelization and ameliorate wound contraction rates. Polyphenols have a role in the hemostasis phase due to some polyphenol moieties (catechol or pyrogallol groups), which can interact with serum proteins in the blood, forming complexes that can stop bleeding by creating a physical barrier [33]. These hemostatic effects can also be due to the antibacterial properties of polyphenols. 

#### 3.1.1. Flavonoids

**Figure 2:**
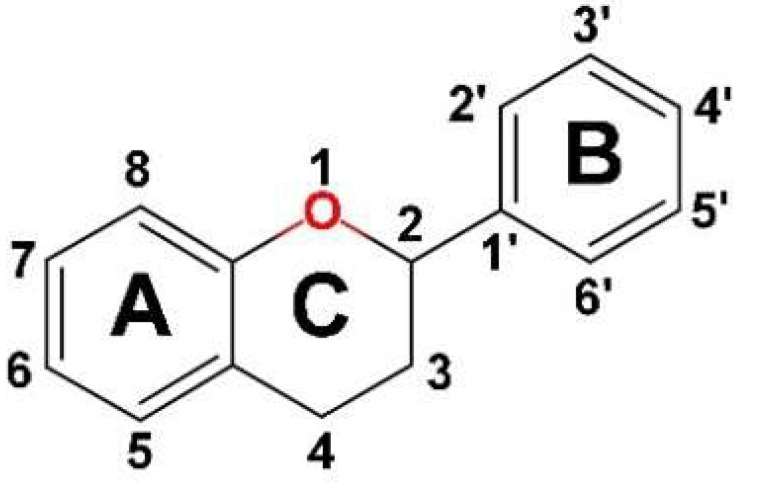
The basic chemical structure of flavonoids with two benzene rings (labeled A and B) and a heterocyclic pyran ring (labeled C).

Flavonoids, important bioactive phenolic compounds abundant in plants, have chemical structures based on the fifteen-carbon skeleton, a common basic structure of diphenylpropanes (C6-C3-C6), with two benzene rings (A and B) linked by a heterocyclic pyran ring (C) (Figure 2). They are subdivided into 12 major classes based on the level of oxidation and pattern substitution of the pyran ring, of which the more significant are flavones (apigenin, luteolin), flavonols (quercetin, kaempferol), flavanones (hesperidin, naringenin), anthocyanidins, isoflavones (genistein), flavan-3-ols (flavanol) (catechin) [30,34,35].

Tannins, an important group of flavonoid compounds, comprise four main categories according to their chemical structure: condensed tannins (proanthocyanidins), hydrolysable tannins (gallotannins and ellagitannins), phlorotannins and complex tannins (catechin glycosidic linked to gallotannins and ellagitannins). Condensed tannins are oligomers and polymers of flavan-3-ol monomers and their most common basic units are represented by (+)-catechin, (−)-epicatechin, (+)-gallocatechin, (−)-epigallocatechin and (−)-epigallocatechin gallate [30,36].

Flavonoids possess an extensive spectrum of pharmacological activity, including antioxidative, anti-inflammatory, antimicrobial, antidiabetic, antimutagenic, anticarcinogenic properties. They have also demonstrated photoprotective, chemoprotective and wound healing properties, which led to several studies related to potential applications in skin diseases, such as aging and skin cancer. Much research has shown that flavonoids, such as curcumin, hesperidin, luteolin, quercetin, kaempferol, apigenin, rutin, naringin, morin, mangiferin have potential for the management of diabetic wounds through stimulation of ECM synthesis and angiogenesis, and modulation of growth factors (Table 1). Thus, curcumin accelerated the healing process by reducing inflammation and had an apoptotic effect in the early stages of wound healing [28]. Luteolin significantly reduced the expression of inflammatory factors, such as IL-1, IL-6 and TNF-α and reduced the levels of prostaglandin E2, interferon and leukotriene B4 [28]. Also, luteolin down-regulated the expression of NF-kB, while inducing an increase in antioxidative enzyme activity, such as superoxide dismutase 1 (SOD1) and glutathione peroxidase (GSH-Px). An in vivo treatment with kaempferol of excisional wounds in diabetic rats showed remarkable results after 14 days, with a higher healing rate of 92.12% and re-epithelization scores, compared to the control [37]. Flavonoids also regulated MMP-2, MMP-8, MMP-9, MMP-13 through the Ras/Raf/ MEK/ERK, PI3K/Akt signaling pathways in diabetic wounds and activated Nrf2 expression, stimulating diabetic wound healing through cellular stress decrease, acceleration of cell proliferation and neovascularization [28]. 

#### 3.1.2. Non-Flavonoid Compounds

The group of non-flavonoid compounds includes phenolic acids and stilbenes. Phenolic acids are characterized by a phenol ring containing at least one carboxylic acid group and derived from two main phenolic compounds, benzoic acid and cinnamic acid, respectively [30]. Hydroxybenzoic acids containing seven carbon atoms (C6-C1) are the simplest phenolic acids found in nature and occur in their free or conjugated forms. Some representative examples: caffeic, ferulic, p-coumaric and sinapic acids. Hydroxycinnamic acids are characterized by a C6-C3 chain, rarely occur in their free form in plants and are represented by gallic, syringic and vanillic acids [30]. In vivo studies showed several roles of phenolic acids in speeding up the wound healing by blocking leukocytes infiltration, ROS scavenging, improving cell attachment and promoting tissue regeneration even in diabetic wounds [38]. Thus, it was found that gallic acid embedded in glucomannan composite hydrogel diminished inflammatory markers and favored COL aggregation and re-epithelization of diabetic wounds [39], while sinapic acid-loaded hydrogels showed a similar effect on full-thickness excisional wounds in streptozotocin (STZ)-induced diabetic rats to the ointment used as positive control [40].

Stilbenes, derived from the phenylpropanoid pathway, make up a polyphenol group characterized by two phenyl rings connected through a two-carbon methylene bridge (C6-C2-C6). The most studied compound is resveratrol, recognized for its potent antioxidant and anti-inflammatory activities [41]. Both in vitro and in vivo studies in human umbilical vein endothelial cell culture and diabetic rat models showed that a topical administration of pterostilbene exhibited a stronger effect than resveratrol in the suppression of HIF-1α and the normalizing of oxidative stress and, thus, accelerated diabetic wound healing [42].

#### 3.1.3. Other Plant Bioactive Compounds

Triterpenes and triterpenoids (a functionalized form of triterpenes), an important group of terpenoids, contain at least 18 subclasses of compounds, of which the most known are saponins, squalene derivatives and phytosterols [28,43]. Carotenoids and β-carotene as a predominant compound are direct precursors of vitamin A, while tocopherols consisting of α- and γ-tocopherols are the precursors of vitamin E. A broad compound group is represented by alkaloids with caffeine as a major component, having a heterocyclic ring structure with at least one nitrogen atom and exhibiting alkali-like properties. Glucosinolates are negatively charged compounds consisting of an S-linked thioglucose unit, an O-sulfated thiohydroximate group and a highly variable side chain, responsible for different biological properties, such as chemopreventive and anti-inflammatory activity [30]. 

The medicinal plant-derived naphthoquinone plumbagin (5-hydroxy-2-methyl-naphthalene-1,4-dione) isolated from the roots of *Plumbago zeylanica* is a promising compound for diabetic wound healing [33]. Shao et al. (2019) showed that plumbagin promoted the wound closure and contraction of diabetic rat wounds by epithelialization acceleration, COL type I deposition, promotion of insulin secretion and improvement of the antioxidant enzymes’ activity [44]. At the gene level, plumbagin up-regulated the expression of Nrf-2, TGF-β and α-SMA and down-regulated the expression of Keap1 in diabetic rats [44]. Also, plumbagin increased EGF, VEGF and FGF, decreased MMP-2, COX-2, iNOS, CD8, CD163 and NF-kB p65 and suppressed IL-6 and IL-1β [33]. 

Nerolidol or peruviol (3,7,11-trimethyl-1,6,10-dodecatrien-3-ol) is a naturally occurring sesquiterpene alcohol found in the essential oils of different types of aromatic medicinal plants. Several biological and pharmacological effects have been reported, such as anti-inflammatory, antimicrobial, antibiofilm and repellent properties [45]. 

Ginsenosides isolated from *Panax ginseng* have a four-ring structure with a steroidal body and sugar moieties. It has been demonstrated that ginsenoside Rb1 displayed a promising effect in diabetic chronic wound healing due to an in vitro significant increase in cell proliferation, COL synthesis and up-regulation of VEGF, TGF-β1 and TIMP-1 in fibroblast culture obtained from patients with DFUs [46]. In addition, in vivo treatment with ginsenoside Rb1 confirmed the role of secreted VEGF in the formation of thick granulation tissue with more new blood vessels, while TGF-β1 and TIMP-1 increased COL synthesis [46]. Similarly, notoginsenoside R1 from *Panax notoginseng* facilitated wound healing in diabetic rats by elevating COL synthesis, increasing ECM secretion, accelerating the wound closure rate, up-regulating the expression of CD31 and down-regulating the expression of caspase-3. An administration of notoginsenoside R1 promoted down-regulation of MMP-3, MMP-9, IL-1β and IL-6 and up-regulation of TIMP1 and TGF-β1 [46]. 

**Table 1:** Action of polyphenols in diabetic wound healing.

Polyphenols Class	Compound	Activity	Reference
Flavonoids	*Luteolin*	In vivo decrease in blood glucose levels, accelerated skin wounds’ re-epithelization in diabetic rats by inhibiting the inflammatory cell infiltration, decreasing IL1-β, IL-6, TNF-α expression, and reducing oxidative stress, down-regulated NF-kB and up-regulated SOD1 and glutathione peroxidase (GSH-Px) expression mediated by p-Nrf2; In vivo intraperitoneal administration treated diabetes-associated wounds by targeting NF-kB/MMP-9 axis and Nrf2-mediated antioxidant system.	[46]
	*Quercetin*	In vivo modulated fibroblast activity, up-regulated VEGF and TGF-β1 in diabetic scars; In vivo oral application increased COL synthesis, deposition and orientation and decreased inflammatory cytokines (IL1-β and TNF-α) in rat diabetic wounds; In vivo topical administration modulated cytokines and GFs, and inhibited inflammatory reactions in rat diabetic wounds; promoted macrophages’ M1-M2 phenotype switch during wound healing in diabetic mice.	[33,35,46]
	*Rutin (quercetin-3-O-rutoside*	In vivo prevented oxidative stress and inflammatory response, improving wound healing in hyperglycemic rats; In vivo decreased the number of inflammatory cells; stimulated Nrf-2 activity and antioxidant enzymes (SOD1 and GSH-Px) expression; In vivo down-regulated IL-1β, IL-6, TNF-α, NF-kB, MMP-2, MMP-9, TGF-β1, VEGF expression levels; In vivo intraperitoneal administration elevated neurogenic-related protein expression.	[46]
	*Myricetin*	In vitro prevented cellular oxidative stress by regulating antioxidant enzymes; In vitro enhanced pro-COL I and III levels, inhibited MMP-1, MMP-2 and MMP-9 synthesis, increased TIMP1/MMPs ratio by enhancing TIMP-1 mRNA expression, suppressed catalase (CAT) and SOD1 in diabetic fibroblasts.	[46,47]
	*Icariin*	In vivo anti-inflammatory and pro-angiogenic activities in diabetic rats by down-regulating NF-kB, TNF-α, MMP-2, MMP-9 levels and increasing IL-10 and CD31 levels; In vivo topical administration stimulated normal ECM formation in the healing tissue by increasing the relative COL deposition.	[46,48]
	*Vicenin-2 (VCN-2)*	In vivo inhibited oxidative and inflammatory stress in a dose-dependent manner, stimulating wound healing in STZ-induced DM rats; In vitro increased cell proliferation, reduced inflammatory cells, down-regulated proinflammatory cytokines (IL-1β, IL-6, TNF-α), mediators (iNOS, COX2) and nitric oxide (NO) expression via NF-kB pathway; improved epithelialization and remodeling; stimulated fibroblast proliferation and migration, neoangiogenesis and wound contraction, down-regulated MMP-9, VEGF and TGF-1β levels via HIF-1α pathway; In vivo topical administration reduced food and fluid intakes, decreased blood glucose level and increased insulin level, body weight and percentage of wound closure.	[46,49]
	*Mangiferin*	In vivo topical administration inhibited oxidative stress, decreased the wound area and increased skin thickness; enhanced EGF, FGF, Nrf-2, TGF-β, VEGF and PI3K expression and decreased MMP-2, TNF-α and NF-kB p65 expression in diabetic wound; reduced the inflammatory phase in hyperglycemic conditions.	[46,50]
	*Curcumin*	In vivo topical administration accelerated re-epithelialization rate, accelerated wound closure through down-regulation of TNF-α, IL-1β and MMP-9 levels, up-regulation of IL-10 level and elevation of SOD, CAT and GSH-Px activity, improved thick granulation tissue formation, COL synthesis, deposition and orientation in rat models of diabetic ulcer and wound.	[46,51]
	*Kaempferol*	In vivo topical agent with 92.12% wound healing rate in diabetic rats.	[37]
	*Epigallocatechin-3 gallate*	In vivo enhanced wound healing through acceleration of re-epithelization and angiogenesis, reduced cytokines level and inhibited macrophage accumulation, inflammation response and Notch signaling in diabetic mouse wounds.	[52]
	*Hesperidin*	In vivo accelerated angiogenesis and vasculogenesis via up-regulation of VEGF-C, TGF-β, Ang-1/Tie-2 and Smad-2/3 mRNA expression, increased COL deposition and suppressed IL-6 and TNF-α inflammatory mediators, enhancing wound healing of chronic DFUs in STZ-induced Sprague Dawley rats.	[53]
	*Genistein*	In vivo subcutaneous administration modulated oxidative stress, improved angiogenesis by FoxO1 and iNOS suppression; oral administration supported wound healing, lowered oxidative stress and inflammation in diabetic mice wounds.	[35]
	*Puerarin*	In vitro down-regulated inflammatory cytokines expression by inhibition of MAPK and NF-κB inflammatory signaling pathways in high-glucose cell culture; improved polarization to M2 macrophages at cellular level.	[54]
Stilbenes	*Resveratrol*	In vitro exerted antioxidant effect and inhibited TNF-α and NF-kB.	[55]
Phenolic acids	*Ferulic acid*	In vivo inhibited lipid peroxidation, increased CAT, SOD and glutathione expression, elevated NO and serum zinc and copper, improving the healing process in diabetic ulcers.	[56]
	*Syringic acid*	In vivo topical administration improved wound healing by promoting cell migration and proliferation in STZ-induced diabetic rats; significantly reduced MMP-2, MMP-8 and MMP-9 levels, up-regulated TIMP-1 and TIMP-2 levels; elevated COL I, CD31, CD68, α-SMA, TGF-β1 and VEGF content in diabetic wounds.	[46,56,57]
	*Chlorogenic acid*	In vivo stimulated COL production, reduced the level of oxidative and inflammation markers (MDA/NO), increased GSH level, maintained SOD/CAT level, accelerating wound healing in STZ-induced diabetic rats.	[58]
	*Gallic acid*	In vivo ROS scavenger, exerted antioxidant activity, promoting wound healing in a diabetic mouse model.	[59]
Tannins	*Tannic acid*	In vivo antioxidant, hemostatic, anti-inflammatory, antimicrobial activity useful in skin wounds and ulcers.	[58]
Terpenes	*Kirenol*	In vivo exerted anti-inflammatory, antioxidant and wound healing activity by regulation of MMP-2 and MMP-9 expression, inhibition of NF-kB, COX-2 and iNOS expression and MDA content, elevated antioxidant enzymes activity, favoring angiogenesis and formation of granulation tissue in STZ-induced diabetic rats.	[46]
Alkaloids	*Berberine*	In vivo topical application accelerated novel ECM synthesis and wound healing process through modulation of TrxR1 and its downstream JNK signaling, expression of MMP9 and TIMP1, up-regulation of TGF-β1, resulting in promotion of fibroblast proliferation and inhibition of oxidative stress and apoptosis in HFD- and STZ-induced diabetic rats.	[46,60]

### 3.2. Natural Polymers

Polysaccharides, also named glycans, comprise monosaccharides and their derivatives linked by glycosidic bonds in long polymeric chains [30]. They are categorized into monopolysaccharides (one type of monosaccharide molecule) and heteropolysaccharides (two or more monosaccharide molecules). Also, polysaccharides differ in their molecular chain length, the degree of chain branching and their positive or negative charge [17]. Polysaccharides elicit promising medical applications due to their extensive obtainability, biodegradability and innocuousness. Moreover, proteins derived from animal ECM are natural polymers of great interest for the development of biocompatible and biodegradable hydrogels.

#### 3.2.1. Cellulose

Cellulose, the most abundant biological polymer, is a polysaccharide made up of D-glucose units connected by β-(1 → 4)-glycosidic linkages. It has a high absorbent capacity, which makes it very effective for intermediate to severe exudate wounds.

Carboxymethyl cellulose (CMC) is a polysaccharide synthesized in alkali conditions for hydroxyl groups’ activation by etherification with monochloroacetic acid, acting as a hydrophilic, biocompatible and biodegradable material that stimulates fibroblast proliferation and migration, being recommended in diabetic wound healing. 

Bacterial cellulose (BC), a type of nano-polymer produced both by Gram-negative bacteria (*Komagataeibacter xylinus*, *Komagataeibacter intermedius*, *Agrobacterium, Azotobacter*, *Achromobacter* spp.) and Gram-positive bacteria (*Komagataeibacter hansenii*) comprises glucose as monomers bonded by β-1,4-glucopyranosyl links. BC has excellent processing properties and is suitable for use as a wound dressing due to its water content (close to 99%), high tensile strength, good thermal stability and good elasticity [61]. In addition, BC maintains a moist wound environment and controls wound exudate and has good biocompatibility and biodegradability. Also, BC possesses unique physical and chemical properties, such as an ultrafine nanofiber network and high crystallinity and water retention capacity [62]. It was reported that the BC-based dressings require specific treatments (e.g., fusidic acid) to ensure their efficacy in wound healing or antimicrobial capacities, but this treatment may cause allergic reactions. The *K. xylinus* strain is commonly used in the production of BC-based dressings for diabetic wound healing [63]. The properties of BC produced by *K. intermedius* showed dramatic differences from that produced by *K. xylinus* [61]. There is a lack of knowledge regarding BC produced from *K. intermedius* for treating diabetic wounds, especially without modification or addition of antimicrobial substances [61].

#### 3.2.2. Alginate

Structurally, alginate, a natural anionic biopolymer, is a linear hydrophilic polysaccharide consisting of D-mannuronic acid (M) and L-guluronic acid (G) units. The units are present in the form of blocks with a variable M/G ratio [63], either as continuous G chain segments (GGGG), M chain segments (MMMM), or alternating MGMG chain segments [1,64]. It is considered that only the G unit is involved in multivalent ion-induced cross-linking of alginate, and a high G content improves the mechanical strength of alginate-based hydrogels [1]. Alginate has valuable biological properties, such as stopping bleeding, moisturizing and allowing gas exchange [14].

#### 3.2.3. Chitosan (CS)

CS, a natural linear copolymer and the only alkaline polysaccharide in nature, is a product of chitin N-deacetylation, which consists of glucosamine and N-acetylglucosamine units. The great versatility of CS has been reported due to its derivatives resulted through the functionalization of its hydroxyl and amino groups by carboxymethyl, carboxyethyl, carboxybutyl, succinyl, acyl, or 5-methyl pyrrolidone moieties [65]. CS has many important biomedical characteristics, such as excellent biocompatibility, biodegradation (could be completely degraded within 14 days after being implanted in animals), non-toxicity, adhesion, inertness, non-antigenicity, antioxidative and good hemostatic properties and wound healing characteristics [1,65]. The cationic nature of chitosan ensures its antimicrobial activity against a wide range of microorganisms (*Escherichia coli*, *Staphylococcus aureus*, *Salmonella typhimurium* and *Listeria monocytogenes*) due to which CS is widely used as wound dressings for diabetics [1,66]. CS mimics the normal ECM environment, induces COL and elastin secretion by promoting fibroblast proliferation and stimulates the growth of granulation tissue and tissue repair [1]. 

#### 3.2.4. Hyaluronic Acid (HA)

The natural polysaccharide HA is the simplest non-sulfated glycosaminoglycan (GAG) (a class of negatively charged polysaccharides) and the main component of the ECM. HA is biodegradable, non-immunogenic, biocompatible with minimal toxicity and has a high capacity for water absorption and water retention. Also, HA actively participates in all stages of wound healing: inflammation, re-epithelization, granulation tissue formation, proliferation and remodeling [12,67]. HA’s action on intracellular signaling pathways mediated by cellular receptors, such as receptor differentiation cluster 44 (CD44), HA-mediated motor receptor, HA endocytosis receptor and lymphatic vessel endothelial cell receptor 1, has been reported, and it decreased the inflammatory response, favoring the tissue repair. This was supported by observations made upon stimulation of anti-inflammatory cytokines IL-2, IL-10 and TGF-β through Foxp3 expression in regulatory T cells [64]. HA was also found to promote angiogenesis and endothelial cell migration by modulation of the MAPK/ERK signaling pathway after binding to CD44 [67]. Recently, HA’s involvement in macrophage activation and polarization into M1 pro-inflammatory or M2 anti-inflammatory phenotypes was demonstrated [1]. This observation allowed for a novel therapeutic strategy using HA to modulate local immunity by favoring polarization to the M2 phenotype in diabetic wound healing. 

#### 3.2.5. Other Polysaccharides

Hsian-tsao polysaccharides, the major functional component in *Mesona procumbens* Hemsl., have strong antioxidant and anti-inflammatory effects. It was reported that Hsian-tsao polysaccharides displayed a promising effect on wound healing in diabetic wounds by decreasing crust and improving the formation of thick granulation tissue with more new blood vessels, and re-epithelialization [46]. In addition, they up-regulated IL-8, TIMP-1, VEGF, MIP-2 and MCP-1 and down-regulated MMP-2 and MMP-9, and suppressed MG-triggered protein glycation and ROS accumulation [68]. Also, they enhanced the methylglyoxal-impeded phagocytosis of *Staphylococcus aureus* and *Pseudomonas aeruginosa* driven by macrophages, which improved the impaired wound healing. It has been shown that polysaccharides were more effective than crude extracts in regulating the factors associated with diabetic wound repair [46].

#### 3.2.6. Collagen

COL is the main protein found in the connective tissue and the most important polymeric component of ECM. It has many functions, including providing tissue strength and structural stability, possesses flexibility and thermal and enzymatic stability, directs cell adhesion and migration, promotes protein secretion and regulates cellular growth and metabolism during development and repair [69]. Also, COL is biocompatible, biodegradable and hemostatic, all important features for wound healing. Among the 28 types of identified and described COL, in the skin, there are mainly COL types I (70%), III (15%) and V. COL plays a significant role in each of the four stages of wound healing. Due to injury, COL exposure activates the clotting cascade during the hemostatic and inflammatory stages of wound healing, resulting in a fibrin clot that stops the initial bleeding [69]. Also, COL activates microRNA signaling pathways to promote the development of a macrophage phenotype that is anti-inflammatory and pro-angiogenic. During wound healing, COL type I replaces COL type III, which is first generated in considerable amounts, creating the scar.

#### 3.2.7. Gelatin

Gelatin (Gel) is the product of partial denaturation and hydrolysis of COL and is widely used in wound healing applications due to its hemostatic properties, good biodegradability and histocompatibility. Gel, generated by the thermal denaturation of COL, has lower immunogenicity than COL and is less likely to cause an immune response in the body [1]. Due to the presence of the arginine-glycine-aspartate (RGD) cell binding sequence and MMP reactive peptide sequence retained in the Gel fabrication process, it promotes cell adhesion mediated by integrins [70].

## 4. Recent Development of Hydrogels Encapsulating Small Molecules for Healing of Diabetic Wounds 

**Figure 3:**
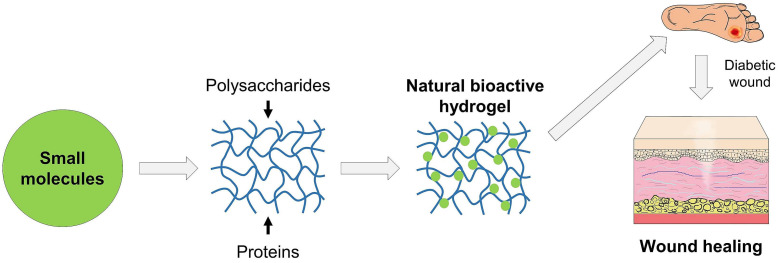
Schematic representation of fabrication of natural bioactive hydrogels, consisting of natural polymeric networks of polysaccharides and proteins loaded with small molecules for their use in diabetic wound healing.

Natural polymer hydrogels are polymeric networks loaded with natural bioactive compounds that can act as good wound dressings due to their versatile physicochemical properties, represented mainly by a high water absorption capacity. Moreover, they provide a temporary structure facilitating cell infiltration and proliferation, and due to their biodegradability, they are gradually replaced by new ECM [71]. The stretching properties confer to hydrogels good adherence to tissue wounds of different shapes. Natural polymer hydrogels loaded with various bioactive compounds provide not only good biocompatibility, but also antibacterial and antioxidant properties and modulation of inflammation at the wound situs to a higher extent compared to traditional dressings, affording promising future applications in diabetic wound healing [17,72] (Figure 3).

Functional hydrogels have been developed and evaluated to overcome the wound infections of diabetic ulcers:

- Antibacterial hydrogels loaded with antibiotics or drug-like components to control bacterial growth;
- Antifouling hydrogels by creating superhydrophilic or more rarely superhydrophobic surfaces, which can reduce bacterial attachment and biofilm formation [22];
- Antioxidant hydrogels loaded with antioxidant scavengers or drugs, such as curcumin and gallic and tannic acid, to improve the inherent antioxidant properties of the constituent macromolecules against ROS formation [73].

### 4.1. Polysaccharide-Based Hydrogels

Various outstanding features of polysaccharides can be used to fabricate biomimetic and multifunctional hydrogels as efficient wound dressings. These hydrogels mimic the natural ECM, are biodegradable, biocompatible and non-toxic, stimulate cell proliferation and have renewability and water-retention abilities. Polysaccharide-based hydrogels have exceptional physicochemical properties and unique therapeutic interventions owing to distinctive architectures and an abundance of functional groups. Multifunctional polysaccharide-based hydrogels are promising for infected and diabetic wound healing. Hydrogels designed using polysaccharides can effectively protect wounds from bacterial attack [17].

#### 4.1.1. Cellulose-Based Hydrogels

Due to their low mechanical strength, cellulose-based hydrogels are usually blended with other polymeric materials, and chemically modified for enhanced wound healing action. Even if cellulose-based hydrogels have revolutionized the design of antibacterial dressings, intensive research is still needed to produce more advanced hydrogels composed of cellulose that can combat the rise of antibiotic-resistant strains and aid in the development of biofilms in wound regeneration [17]. Thus, CMC plant fibers were used to develop hydrofiber dressings that produce a soft gel whenever they contact wound exudates [65]. A combination of CMC and carbomer 940 containing a polysaccharidic extract of *Periplaneta americana* was very efficient in M2 macrophage polarization, COL deposition, angiogenesis and accelerating wound healing in diabetic rat wounds [74]. CMC/sericin-based hydrogels with intrinsic antioxidant, anti-inflammatory and antibacterial properties promoted re-epithelization of diabetic rat wounds [75]. Cellulose-derived cotton gauze dressings have been traditionally used in clinical settings and have proved to have the capability to accelerate the wound healing process by a controlled release of several growth factors at the injury site [65]. Thus, the release of basic fibroblast growth factor (FGF), EGF and phosphodiesterase growth factor promoted the migration and proliferation of fibroblasts and inhibited bacteria proliferation in the wound. A hydroxymethyl cellulose-based hydrogel also containing uniformly dispersed MnO_2_ nanosheets was proved to act as an ROS scavenger, converting them to O_2_, needed in different stages of wound healing to lower in situ inflammation, while an addition of silk fibroin inhibited MMPs gene expression for a synergistic promotion of matrix remodeling [76]. To reinforce the structural and mechanical properties of hydrogels mimicking the microenvironment of natural ECM, a BC matrix was impregnated with carbon nanotubes and showed higher antibacterial activity compared to the ointment used as positive control and faster healing of diabetic wounds in an STZ-induced model in BALBc mice, compared to the untreated control [77]. 

#### 4.1.2. Alginate-Based Hydrogels 

Alginate hydrogels are recommended as dressings for diabetic wound healing due to their unique advantages, compared to other hydrogels, i.e., they are highly hydrophilic and very useful for excessive exuding wounds, and they can change their fine porosity in contact with exudate, increasing their volume, and do not present tissue adhesion, allowing a safe repair process [1]. 

An injectable hydrogel with therapeutic efficiency was made from sodium alginate and chondroitin sulfate using a solvent casting method and enriched in curcumin to exert ROS scavenging ability, biocompatibility and antibacterial activity in an excisional wound model developed in diabetic rats [78]. 

In a clinical study, sodium alginate hydrogel enriched with fatty acids and vitamins A and E proved its efficacy in foot wound healing of diabetic patients with a 22.2% reduction of the lesion area, the pressure ulcer scale for healing (PUSH) score dropping from 9.8 to 6.6, or even complete wound healing [79]. The great potential of alginate dressings combined with EGF was found in a randomized controlled trial with 18 patients with refractory wounds as increased proliferation and differentiation of epidermal stem cells, compared to an EGF-alone treatment [80].

Currently, Purilon^R^ Gel and Nu-Gel^TM^ are the commercialized alginate saline gels for facilitating natural autolytic debridement and faster healing of leg ulcers and non-infected DFU [81].

#### 4.1.3. CS-Based Hydrogels

CS hydrogels have excellent antibacterial properties and good biocompatibility and biodegradability, and several combinations with natural bioactive compounds were designed to demonstrate improved functions during diabetic wound treatment (Table 2). Thus, CS-based hydrogel containing a flavonoidic extract of *Passiflora edulis* Sims leaves showed stimulation of the antioxidant defense system due to the controlled release of bioactive compounds, enhancing wound healing in diabetic rats. However, a film covering the wounded site for 14 days can lead to hypoxia and high oxidative stress with increased lipid peroxidation [82]. CS-based hydrogel impregnated with nerolidol promoted significant wound healing [46]. It has been reported to have a diabetic ulcer healing effect within 14 days of treatment with nerolidol-functionalized gold nanoparticles ointment in albino rats, compared with the untreated control group [83]. An injectable hydrogel was developed as CS-CMC-g-PF127, loaded or not with Cur, presenting viscoelastic behavior, good swelling properties, a controlled release profile, thermal stability and crystalline characteristics [84]. The Cur-loaded CS-CMC-g-PF127 injectable hydrogel exhibited fast wound repair potential by the stimulation of the cell migration and proliferation at the damage site and by providing a sustained drug delivery platform for hydrophobic moieties [84]. CS hydrogels encapsulating quercetin or tannin as bioactive compounds significantly promoted the wound healing process in diabetic wounds [85]. 

A clinical trial of Velazco et al. (2012) reported the application of completely biodegradable CS-based dressings on patients with diabetic foot complications [86]. After 8 days of treatment, the granulation process began and the size of the lesion decreased after 22 days, while total closure of the ulcer was observed after 45 days. 

There is already a market product ChitoHeal based on CS, which proved in clinical trials several advantages in burns, cuts, scratches and DFU treatment due to its biocompatibility, being effective in scar and healing rate reduction [87].

**Table 2:** Activity of chitosan-based hydrogels in diabetic wound healing.

Hydrogel Type	Experiment Type	Activity	Reference
Quaternized CS/tannic acid hydrogels	STZ-induced diabetic rat model	Good injectability and self-healing, cytocompatibility, hemostatic capability and biodegradability, radical scavenging activity, COL deposition, no scar formation, skin regeneration.	[88]
CS-puerarin hydrogel	STZ-induced diabetic rat model	Promoted diabetic wound healing and accelerated angiogenesis, inhibition of the miR-29 mediated inflammation response.	[1,89]
Sulfated chitosan (SCS)-doped COL type I (Col I/SCS) hydrogel	STZ-induced diabetic rat model	Reduced inflammation through minimizing macrophages’ polarization into M1 phenotype, decreased production of pro-inflammatory IL-6 and increased production of anti-inflammatory cytokines IL-4, TGF-β1 in chronic diabetic wounds; stimulated COL synthesis, angiogenesis and cell migration for wound closure in diabetic wounds.	[1,90]
Apigenin loaded gellan gum-CS (GGCH) hydrogel	STZ-induced diabetic rat model	Increased level of SOD, GSH, CAT, protein content in granuloma tissue; biocompatibility, biodegradability, moist nature, antioxidant effectiveness; increased hydroxyproline level and collagen turnover; decreased epithelialization period; higher wound healing in diabetics.	[91]
CS/ HA-based hydrogel with MOF-loaded lipoic acid	In vitro and in vivo analysis in diabetic Sprague Dawley rats	Antibacterial activity and antioxidant performance, promoted cell proliferation and migration, wound healing process, better granulation tissue formation and more COL deposition.	[92]
Bio-multifunctional benzaldehyde-terminated 4-arm PEG (4-arm-PEG-CHO)/carboxymethyl CS (CMCS)/basic fibroblast growth factor (bFGF) hydrogels (BP/CS-bFGF)	STZ-induced diabetic rat model	Strong wet-tissue adhesion, self-mending fast hemostasis capacity, excellent biocompatibility, antibacterial property, increased production of Ki67, promoted the generation of epithelialization and COL, induced formation of hair follicles, enhanced neovascularization by up-regulating the production of CD31 and CD34.	[93]
CS hydrogels functionalized with either unfractionated heparin or bemiparin (a low molecular weight heparin, LMWH)	STZ-induced diabetic rat model	Accelerated inflammation, improved the epithelization process, formation of high-quality cicatricial tissue, improved diabetes-associated impaired wound healing.	[94]

#### 4.1.4. HA-Based Hydrogels

HA-based hydrogels are promising tools in wound healing applications due to their composition, structure and size. Due to HA functional groups, including carboxylic acids (–COOH), primary and secondary hydroxyl groups (–OH) and *N*-acetyl groups, useful cross-linking can increase the mechanical strength of the hydrogel. Also, the combination of HA hydrogel with small molecules can improve its physical and chemical characteristics, like biocompatibility, biodegradability, mass transferability and antibacterial activity [95] (Table 3). 

### 4.2. Protein Hydrogels

#### 4.2.1. Collagen-Based Hydrogels

COL-based hydrogels are characterized by a fibrillar network forming a porous structure, biomimetic to initial ECM, acting to stimulate cell migration and colonization and new ECM constituent synthesis for deep and surface wound healing. Despite these advantages, COL-based hydrogels do not present antibacterial and antioxidant activity for more efficient skin wound repair [69]. However, Apligraf^®^ (Organogenesis Inc., Canton, MA, USA) and Dermagraft^®^ (Organogenesis Inc., Canton, MA, USA) are two commercialized COL-based hydrogels approved by the Food and Drug Administration (FDA). The use of Apligraf in a randomized controlled trial on neuropathic DFU patients promoted cell proliferation and 51.5% of cases reached complete wound closure in 12 weeks [100]. There is currently research being conducted on novel compositions of COL-based hydrogels loaded with bioactive compounds that can improve the biological properties required for skin wound treatment, in particular for diabetic patients with chronic wounds [72,101]. Clinical studies indicated that pre-vascularized COL/fibrin hydrogels increased hypodermis thickness and accelerated the wound healing process in DFU patients [102]. Ulcers presented higher healing rates compared to standard treatment with saline gauze and healed completely after 12 weeks of treatment with COL/CS hydrogels in neuropathic DFU patients [102].

#### 4.2.2. Gelatin-Based Hydrogels

Narisepalli et al. (2023) developed a Gel-based biodegradable hydrogel embedding pentacyclic triterpene asiaticoside nanoparticles for diabetic wound healing. In vitro cell culture studies demonstrated significant proliferation and migration of fibroblasts, while application to the wounds of diabetic rats improved wound healing in terms of improving COL biosynthesis, up-regulating the COL type I protein level and enhancing α-SMA gene expression, compared to control untreated groups [103]. Recently, Gel-based thermoresponsive injectable hydrogels with antioxidative, antiinfection, antibacterial, biocompatibility and tissue regeneration characteristics have become of great interest as potential candidates for effective regeneration of diabetic wounds. Liu et al. (2018) developed a thermosensitive hydrogel containing Cur nanoparticles loaded in Gel microspheres, a system for efficient, safe and fast healing of standardized skin wounds created in STZ-induced diabetic mice [104]. Using an emerging technology of 3D bioprinting, Xia et al. (2022) designed GelMA hydrogel loaded with Cur, an antioxidant and anti-apoptosis compound, to regulate the microenvironment of diabetic wounds [105]. This hydrogel encapsulating adipose-derived stem cells (ADSCs) could repair full-thickness skin defects of diabetic nude mice by significantly down-regulating AGEs and ROS production, reducing ADSC apoptosis, promoting angiogenesis and wound re-epithelialization, and targeting the AGEs/AGER/p65 pathway.

#### 4.2.3. Other Protein Hydrogels

Recently, Bai et al. (2023) developed a sericin-based hydrogel containing injectable platelet-rich fibrin (i-PRF), a biocompatible material with anti-inflammatory properties. i-PRF is rich in growth factors, platelets, leukocytes and fibrin, which play important roles in different wound healing stages, being useful in diabetic wound repair [103]. The controlled release of i-PRF-derived bioactive growth factors from the hydrogel reduced inflammation levels, increased COL synthesis, promoted angiogenesis and led to wound healing in diabetic nude mice [106].

## 5. Conclusions and Future Remarks

In conclusion, there are remarkable effects of the hydrogels enriched with natural bioactive compounds for improving diabetic-related chronic wound healing. They could provide novel therapeutic strategies to prevent limb amputation and increase the quality of life in diabetic patients. In vitro and in vivo studies in rat models of diabetic wounds demonstrated the positive effect of polyphenols as natural bioactive compounds in all stages of healing, alone, complementary or even synergistic to the functional properties of natural polysaccharidic or protein hydrogels. Limitations of clotting, oxidative stress and inflammation, cell proliferation and tissue remodeling found in natural polymeric hydrogels were improved after small molecules encapsulation, as found by specific markers analysis at the gene expression and protein secretion levels. Future studies will contribute to further improving the composition and structure of biocompatible functional hydrogels and help us face the challenges of revealing the complex mechanisms of diabetic wound healing.

## Figures and Tables

**Table 3:** Activity of hyaluronic acid-based hydrogels in diabetic wound healing.

Hydrogel Type	Experiment Type	Activity	Reference
Paeniflorin-loaded HA-based hydrogel	Ex vivo and in vivo experimental approaches in diabetic mice model	Stimulated transition of macrophages from M1 pro-inflammatory phenotype to M2 anti-inflammatory/pro-healing phenotype, lowered inflammation and promoted COL synthesis, new blood vessel formation, re-epithelialization of cutaneous wounds.	[96]
Hyaluronan/COL-based hydrogels containing high-sulfated hyaluronan	In vitro and in vivo studies in diabetic db/db mice	Reduced inflammation, augmented pro-regenerative macrophage activation, increased vascularization, accelerated new tissue formation and wound closure.	[97]
Nanotechnologically-modified curcumin and EGF encapsulated into HA and CS-based hydrogel	In vitro and in vivo studies in STZ-induced diabetic mice	High antioxidant, anti-inflammatory and migration-promoting effects, improved wound healing by granulation tissue formation, re-epithelialization and skin regeneration.	[98]
Glucose-responsive HA derivate (HAMA-PBA)/catechin (HMPC) hydrogel	In vitro and in vivo studies in diabetic wound model	High antioxidant capability, increased expression of VEGF and CD31, stimulated angiogenesis, decreased inflammatory responses by low IL-6 level and high IL-10 level, fast wound repair in three weeks.	[99]

## Data Availability

Not applicable.
